# Features of rheumatic fever in adult patients in modern Kyrgyzstan

**DOI:** 10.1186/ar3692

**Published:** 2012-02-09

**Authors:** Nazgul A Omurzakova, Aynagul S Djumagulova, Raisa I Rudenko, Kusuki Nishioka, Toshihiro Nakajima

**Affiliations:** 1 National Center of Cardiology and Internal Medicine, Bishkek, Kyrgyz Republic; 2 Institute of Medical Science, Tokyo Medical University, Tokyo, Japan

## Objective

Study of peculiarities of rheumatic fever (RF) in adult patients.

## Materials and methods

We have studied prospectively for 5 years 200 patients (56 men and 144 women) with acute rheumatic fever (ARF in 27) and recurrent ARF (in 173) at the age of 15-40 years (average age ± 24,5 7 years). Clinical and laboratory (ESR, antistreptolysin-O (ASL-O) and CRP) and instrumental (ECG, ECG monitoring daily, 2-D echocardiography color) studies conducted. The diagnosis of ARF was verified according to the WHO diagnostic criteria in the modification of Jones' criteria, AHA (1999) and WHF (2008).

Results: We found that predisposing factors for the development of ARF was the presence of tonzillopharingitis (47.5%), while carriers of group A streptococcus (GAS) was 38.0% among patients examined. Clinical symptoms of carditis with echocardiographic signs of valvulitis occurred in 196 (98.0%) patients. In 54 (27.5%) of them installed valvulitis mitral valve. Valvulitis aortic valve was detected in 24 (12.2%) patients. In 118 (60.2%) patients observed at the same time valvulitis mitral and aortic valves, while in 22 (39.2%) patients are men and 92 (63.8%) patients are women. In 18 (66.6%) patients with ARF was observed mitral valve prolapse (MVP), in 6 (22.2%) were in men, 12 (44.4%) in women. In 9 (4.5%) patients with ARF proceeded pancarditis (endocarditis, myocarditis and pericarditis). Signs of coronaritis with typical anginal pain with ECG signs of ischemia, arrhythmias, heart block were observed in 12 (6.0%) patients with RF. Verification of diagnosis was carried out using the angiography of coronary arteries. The symptoms of coronaritis in this patients disappeared after anti-inflammatory therapy. Polyarthritis with ARF was observed in 40.7% of patients, 25 (14.4%) of patients with recurrent ARF articular syndrome manifested primarily arthralgia. In addition, 6.5% in patients with RF were observed asymptomatic sacroiliitis stage I-II (Dale), 7 of patients are men and 5 of them are women.

## Conclusion

The reducing of clinical manifestations of ARF in adult led to gypo-diagnostics of disease, a consequence of which was the formation of rheumatic heart disease.

